# Lessons learned in infection prevention for Ebola virus disease and the coronavirus disease 2019 (COVID-19) pandemic—Principles underlying prevention

**DOI:** 10.1017/ice.2020.1427

**Published:** 2021-01-11

**Authors:** Erica S. Shenoy, David J. Weber

**Affiliations:** 1 Infection Control Unit, Massachusetts General Hospital, Boston, Massachusetts; 2 Division of Infectious Diseases, Massachusetts General Hospital, Boston, Massachusetts; 3 Harvard Medical School, Boston, Massachusetts; 4 Division of Infectious Disease, School of Medicine, University of North Carolina at Chapel Hill, Chapel Hill, North Carolina; 5 Department of Hospital Epidemiology, UNC Medical Center, Chapel Hill, North Carolina

**Fig. 1.**
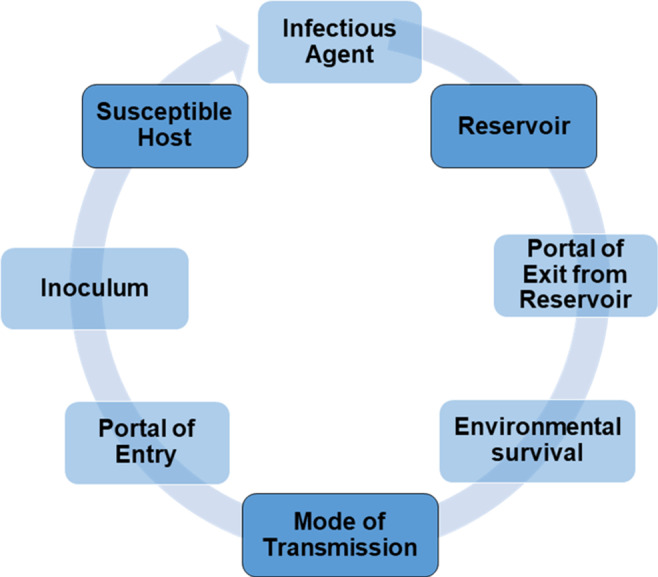
The chain of transmission. Transmission from one individual to another requires completion of each step in the chain of transmission. Beginning with an infectious agent in a reservoir (eg, human, animal, or inanimate object/surface), the infectious agent must exit the reservoir through portal of exit; survive in the environment; and be transmitted by contact, droplet, or airborne routes (or a combination thereof); and enter as susceptible host through a portal of entry (eg, eyes, nose, mouth, wound) at an inoculum sufficient to establish infection.

Delivery of care to patients with highly communicable diseases balances the potential risk of transmission from the patient-to-healthcare personnel (HCP) with the risks to the patient of delayed or reduced access to needed interventions. The risk of transmission to HCP depends on many factors described by the chain of transmission (Fig. 1), including the establishment of a reservoir (human, animal, inanimate environment), exit of the infectious agent from the reservoir and survival in the environment, with transmission by direct or indirect contact, droplet, airborne modes or combinations of these modes, and finally entry of the infectious agent via a portal of entry to a susceptible host at an inoculum sufficient to establish infection. Efforts to prevent transmission in healthcare settings—among patients, visitors, and HCP—are all aimed at interrupting the chain of transmission. These efforts include, in addition to correct and consistent use of personal protective equipment (PPE) plus rapid institution of appropriate isolation precautions as indicated by the mode of transmission, multiple other interventions that minimize the risk of nosocomial transmission. These measures are often framed as part of the hierarchy of controls applied to HCP safety, but they also have applications for reducing overall risk of transmission to patients and visitors.^1^

In this issue of *Infection Control and Hospital Epidemiology*, DiLorenzo et al^2^ report on a survey of policies of Ebola treatment centers with respect to provision (planned or actual) of critical care interventions for patients with viral hemorrhagic fevers (VHFs) such as Ebola virus disease (EVD).^2^ The researchers distributed a 58-item survey to 82 Ebola treatment centers between January 2020 and March 2020, and report on the responses of 17 institutions of which fewer than half had experience caring for patients with VHFs or persons under investigation (PUIs). They used the survey to query institutions on policies in 9 critical care areas: renal replacement therapy, endotracheal intubation and mechanical ventilation, extracorporeal membrane oxygenation, chest compressions, pharmacological cardioversion, electrical cardioversion, defibrillation, cricothyrorotomy, and code status. The survey further inquired about the extent to which staff safety, lack of appropriate technology, lack of clinical guidelines, clinical futility, and limitations of the environment of care, influenced policies regarding provision of care. Most respondents had policies regarding replacement, endotracheal intubutation and mechanical ventilation, and chest compressions, although applications of each varied by patient-level factors. For other interventions, fewer respondents reported having policies, and types of patients (PUI vs confirmed VHF) to whom it would be offered varied. Among the factors influencing decision regarding offering care to either PUIs or confirmed VHF patients, staff safety and clinical futility were reported to influence decisions “somewhat” or “greatly” for most respondents whereas lack of appropriate technology, guidelines, or physical limitations in the environment of care either did not limit care (or minimally limited care) according to most respondents.

The fact that HCP safety has such a prominent impact on decisions to offer particular types of care is not unique to VHFs; it has been a concern raised in the provision of care to patients infected with SARS-CoV-2. Some prominent differences between the two pathogens underly the potential risk of nosocomial acquisition to HCP. Specifically, the primary modes of transmission (EVD primarily through contact and SARS-CoV-2 primarily through droplets), and the ability of individuals to transmit infection while asymptomatic or presymptomatic (not considered likely with EVD but prominent with SARS-CoV-2) are key aspects that inform the infection prevention strategies (Table 1).

**Table 1.** Comparison of Pathogens Primarily Transmitted by Contact with Body Fluids (eg, Ebola virus) Versus Respiratory Droplets and Droplet Nuclei (eg, SARS-CoV-2)

Variable	Ebola Virus	SARS-CoV-2
**Microbiology**		
Year identified	1976	2019
Family	Filaviridae	Coronaviridae
Genome	RNA	RNA
Coat	Enveloped	Enveloped
**Epidemiology**		
Prevalence	Repeated outbreaks	Pandemic
Reservoir	Bats	Bats; research ongoing to identify additional potential reservoirs
Intermediate host	Primates and other animals	None demonstrated
Primary mode of transmission	Direct Contact: Contact with infectious body fluids	Respiratory droplets
Other modes of transmission	Indirect contact (ie, contaminated surfaces, devices), sexual, blood transfusion	Direct and indirect contact (ie, contaminated surfaces, devices)
Basic reproductive rate (R_0_)	1.5–2.0^23^	1.8–3.6^24^
Asymptomatic and presymptomatic transmission	No	Yes
Incubation period	6–12 d (range, 2–21)	2–14 d
Case fatality rate	˜50% (range, 25%-90%)	∼15% among hospitalized patients
Treatment	Monoclonal antibody combination (atoltivimab, maftivimab, and odesivimab-ebgn)	Remdesivir, bamlanivimab
**Infection prevention**		
Nosocomial transmission involving HCP (HCP-to-HCP, HCP-to-patient, patient-to-HCP)	Yes	Yes
Laboratory biosafety level	BSL-4	BSL-2 (routine diagnostic testing); BSL-3 (virus isolation in cell culture)
Survival on surfaces	Hours to a few days	Hours to a few days
Antiseptic	60%–90% alcohol-based product	60%–90% alcohol-based product
Disinfectant	EPA, emerging virus claim (List “N”)	EPA, emerging virus claim (List “N”)
Special handling of used linens, patient waste	Yes	No
PPE worn by HCP (CDC)	1. Single-use (disposable) fluid-resistant gown that extends to at least mid-calf or single-use (disposable) fluid-resistant coveralls without integrated hood 2. Single-use (disposable) full face shield 3. Single-use (disposable) face mask 4. Single-use (disposable) gloves with extended cuffs. Two pairs of gloves should be worn. At a minimum, outer gloves should have extended cuffs.^25^	1. N95 respirator (or equivalent or higher-level respirator) or facemask (if a respirator is not available) 2. Eye protection (ie, goggles or a face shield that covers the front and sides of the face) 3. Single use (disposable), clean, nonsterile gloves 4. Single use (disposable) isolation gown or cloth gown.^26^
Pre-exposure prophylaxis	Vaccine	Vaccine
Postexposure prophylaxis	None approved for postexposure prophylaxis	None

Note. BSL, biosafety level; EPA, US Environmental Protection Agency; HCP, healthcare personnel.

The data available for VHFs and SARS-CoV-2, however, demonstrate that the major risks to HCP stem from failure to identify patients at entrance to a healthcare facility as possibly infected and to isolate them appropriately, from failure to utilize personal protective equipment (PPE) correctly especially during donning and doffing, and from inadequate PPE due to shortages. These same challenges have been present during the COVID-19 pandemic. Additionally, HCP-to-HCP spread of SARS-CoV-2 has been linked to lapses in masking and distancing when masks are removed to eat or drink (eg, in break rooms and at nursing stations) and when physical distancing is not maintained. Acquistion by HCP has then led in some cases to transmission to patients with propagation of transmission.^3^

The primary intervention to reduce risk of nosocomial transmission relies on early identification of PUIs and intiation of isolation. For both EVD^4^ and SARS-CoV-2,^5–8^ failures at this critical juncture have resulted in exposures to HCP and transmission events. Although self-contamination and cross contamination are concerns with SARS-CoV-2 and careful doffing and use of hand hygiene must be emphasized, transmission directly attributable to doffing failure has not been documented. One SARS-CoV-2 serological study failed to identify an association between positive serology and care of patients with COVID-19; however, these researchers did note a strong association of living in a household with an individual with suspected or confirmed SARS-CoV-2 infection.^9^ Another serological study of HCP noted lower prevalence of SARS-CoV-2 antibodies among HCP who reported consistent use of a face mask when caring for patients.^10^

In contrast, due to contact with blood and body fluids as the primary mode of transmission with EVD, self- and cross-contamination is a priority concern to the extent that extensive training in the use of PPE, careful selection of PPE components and order of doffing, close attention to the design of the physical space where doffing occurs is warranted, and the implementation of a trained observer is recommended by the CDC to ensure each HCP doffs correctly and that instances of possible contamination are identified during the process and mitigated.^11–15^ Use of dedicated HCP with who have trained and exercised in the use of PPE for Ebola is recommended both to the high-risk aspect of doffing PPE while avoiding self-contamination and cross contamination, as well as the fact that the PPE used for EVD and other viral hemorrhagic fevers is not used routinely in most healthcare settings. Adjunctive approaches, such as techniques to visualize contamination^16^ and the use of ultraviolet disinfection of PPE,^17^ have been shown to reduce the transmission risk to HCP. In some settings with EVD, and worldwide with SARS-CoV-2 due to the large-scale global nature of the pandemic with resultant interruption of supply chain, PPE shortages have led to strategies that have included extended use, reuse of PPE following disinfection or decontamination, and use of alternative PPE components that have not been certified, as well as lack of adequate PPE.^18^ HCP-to-HCP transmission of VHFs has been reported.^19,20^ HCP-to-HCP of SARS-CoV-2 infection has been well documented via droplet spread, in part because it can be transmitted from asymptomatic, presymptomatic, and pauci-symptomatic individuals, especially in settings where masking is not present (eg, break rooms).^21,22^ Transmission events are not restricted to HCP interactions in the workplace and are more likely to occur during external social activities where masking compliance between HCP may be reduced.

DiLorenzo et al^2^ demonstrated that HCP safety in provision of critical care to EVD PUIs is informed by assessment of risk of potential for patient-to-HCP transmission. Similar concerns have underscored the COVID-19 pandemic and highlight the importance of multifaceted approaches to interrupting the chain of transmission. Differences between the 2 pathogens, however, specifically the primary modes of transmission and the role of asymptomatic and presymptomatic transmission, underscore differences observed in the overall risk of patient-to-HCP transmission.
